# Complete genomic sequencing and characterization of an ESBL-producing *Escherichia coli* , sequence type 8211, isolated from the fecal material of an Eastern Bongo

**DOI:** 10.17912/micropub.biology.002138

**Published:** 2026-05-20

**Authors:** Elizabeth E. Gorka, Sebastian Christoper Romero, Durward L. Bevis, Nicole A.M. Dutton, Janelle Brandt, Richard W. McLaughlin

**Affiliations:** 1 School of Liberal Arts and Sciences, Gateway Technical College, Kenosha, Wisconsin, United States; 2 Great Plains Zoo & Delbridge Museum of Natural History, Sioux Falls, South Dakota, United States

## Abstract

The Eastern Bongo ( *Tragelaphus eurycerus isaaci* ) is a critically endangered species in the wild. In zoos, animals are common carriers of Extended-Spectrum Beta-Lactamase (ESBL) producing bacteria. The purpose of this study was to determine if the fecal material of an Eastern Bongo from the Great Plains Zoo contained ESBL producing *Enterobacteriaceae* . Bacteria from this animal were inoculated onto HardyCHROM™ ESBL agar. Isolates with a pink colored colony were subject to whole-genome sequencing. One isolate was identified as *Escherichia coli* using PubMLST. A putative conjugative plasmid containing seven antimicrobial resistance genes was also found. A Microscan autoSCAN-4 System was then used to determine antimicrobial susceptibility of the isolate. The sequence type identified was (ST/phylogenetic-group/β-lactamase): ST8211/B1/CTX-M-55 + LAP-2 + TEM-1B.

**Figure 1. Minimum inhibitory concentration of different antimicrobials for E. coli strain Bongo and a putative gene cluster containing several antimicrobial resistance genes found in pLiz1:** A) Minimum inhibitory concentration; R=Resistant; R*=Predicted resistant interpretation; I=Intermediate; S=Susceptible; ESBL=Extended spectrum beta-lactamase. B) Putative gene cluster 1. Class A beta-lactamase (EC 3.5.2.6) => TEM family; 2. Hypothetical protein; 3. Cell division protein FtsI [Peptidoglycan synthetase] (EC 2.4.1.129); 4. Mobile element protein; 5. Class A beta-lactamase (EC 3.5.2.6) => LAP family; 6. Mobile element protein; 7. Transposase InsD for insertion element IS2; 8. Class A beta-lactamase (EC 3.5.2.6) => CTX-M family, extended-spectrum; 9. Tryptophan synthase (indole-salvaging) (EC 4.2.1.122); 10. Aminoglycoside N(3)-acetyltransferase (EC 2.3.1.81) => AAC(3)-II,III,IV,VI,VIII,IX,X; 11. TmrB-like protein; 12. Hypothetical protein; 13. Hypothetical protein; 14. Hypothetical protein; 15. IS2 orf1.

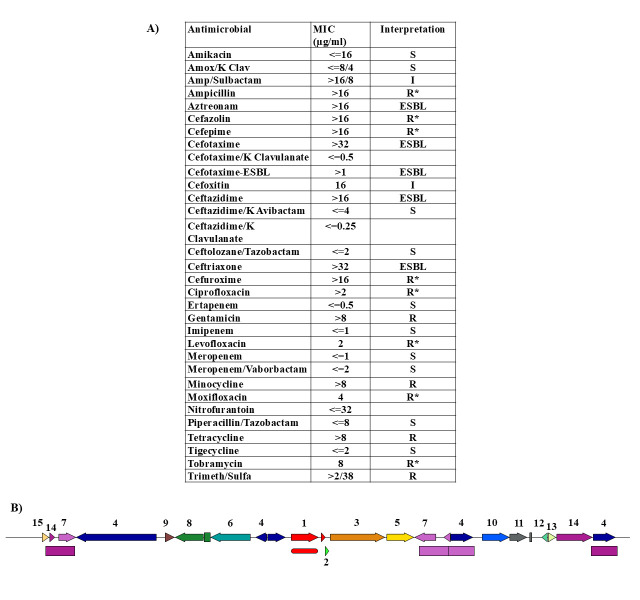



## Description

Bongos are a type of antelope species with spiral shaped horns which are native to sub-Saharan Africa (Rakotoarivelo et al., 2024). The Eastern bongo, ( *Tragelaphus eurycerus isaaci* ), is listed in the IUCN Red Data Book as a critically endangered species, with only 70 to 80 mature individuals remaining in the wild (IUCN, 2017). Their low numbers are mainly due to disease, hunting, loss of habitat, and human encroachment (Percival, 1928; Elkan and Smith, 2013). Interestingly, there is a greater number of animals living in captivity than in the wild.

According to the Centers for Disease Control and Prevention, extended-spectrum β-lactamase (ESBL)-producing *Enterobacteriaceae* are a threat to human health (Centers for Disease Control and Prevention [CDC], 2019). Bacteria that produce ESBLs can hydrolyze first, second, and third generation cephalosporins (Guenther et al., 2010). The ESBL family of enzymes is subdivided into four Ambler scheme classes A-D groups (Ambler, 1980). Two of the most common families are TEM, and CTX-M, in which CTX-M-type is the most prevalent worldwide (Abraham et al., 2018). This family is believed to originate from various *Kluyvera* spp. *Escherichia coli* strains containing these genes have been documented in both farm animals (Gonggrijp et al., 2023), and zoo animals (Isler et al., 2021) in numerous countries. In recent studies, antimicrobial resistant *E. coli* was isolated from captive mammals living in Ecuador, (Medina et al., 2024), black bears living in China (Lei et al., 2025), and an orangutan living in the USA (Smith et al., 2024), thus highlighting the importance of zoo animals as reservoirs for *bla*_CTX-M_ variants.

*bla*_CTX-M_ genes are seldom chromosomally mediated, but are mainly localized on plasmids, such as IncHI2, IncFIA, and IncFIB (Ghosh et al., 2016; Zhang et al., 2019; Hassen et al., 2020; Shawa et al., 2021). Interestingly, IncHI2 plasmids are mostly found in bacteria within the family *Enterobacteriaceae* including *E. coli* . *E. coli* isolates harboring this plasmid type have been documented in cattle (Wang et al., 2023), nursery pigs (Wu et al., 2018), and ducks (Sun et al., 2019) and they often carry antimicrobial resistance genes conferring resistance to β-lactams, and quinolones (Nakayama et al., 2023; García-Fernández and Carattoli, 2010). Many IncHI2 plasmids are conjugative and are thus able to transmit antimicrobial resistance genes to previously susceptible bacterial cells which can lead to the spread of several resistance genes very quickly (Fang et al., 2016).

In this present study, bacteria from the fecal material of an eastern bongo “Bingo” were added to HardyCHROM™ ESBL agar. In total, five isolates with pink colored colonies were screened using whole-genome sequencing (WGS). Bacteria were identified using PubMLST ( https://pubmlst.org/ ) and the results confirmed using average nucleotide identity (ANI). ANI is a reputable gauge of genetic distances between different strains of bacteria (Konstantinidis and Tiedje, 2007). The cut-off value of 95–96% is used as the species boundary (Richter and Rosselló-Móra, 2009). One *E. coli* isolate, strain Bongo, with an ANI value of 96.7% observed when compared to *E. coli* DSM 30083 ^T^ (GenBank accession number CP033092.2), was subject to further analysis *.* Interestingly, strain Bongo contained two plasmid replicons (pLiz1 and pSebas2).

Clermon Typing determined *E. coli* strain Bongo belongs to phylogenetic group B1. This group is often detected in *E. coli* stains isolated from the fecal material of zoo animals living in captivity in the USA (Higgins et al., 2007). Using the BacWGSTdb database (Feng et al., 2021), antimicrobial resistance genes were predicted on both the chromosome and one plasmid replicon (pLiz1). In addition, this database was used to predict the type of plasmid replicons (IncHI2, IncHI2A, IncN) and the sequence type (ST8211). pLiz1 is 255, 514 bp and contains 339 CDS. In total, seven antimicrobial resistant genes ( *aac(3)-IId* , *ant(3”)-Ia* , *bla*_CTX-M-55_ , *bla*_LAP-2_ , *bla*_TEM-1B_ , *floR* , and *inu(F)* ) are plasmid mediated conferring resistant to aminoglycoside, beta-lactam, phenicol, and macrolide classes. Five additional genes ( *aph(3'')-Ib* , *aph(6)-Id* , *mdf(A)* , *sul2* , and *tet(B)* ) are chromosomally mediated conferring resistant to aminoglycoside, macrolide, sulphonamide, and tetracycline classes. Antimicrobial susceptibility was then determined on the isolate. A list of the antimicrobials tested, and the minimum inhibitory concentration (MIC) values are shown in Figure 1A . *E. coli* strain Bongo was identified as potentially encoding an ESBL due to resistant MIC values for aztreonam, cefotaxime, cefotaxime-ESBL, ceftazidime, and ceftriaxone ( Figure 1A ). The NG-Test CTX-M MULTI was also used to verify ESBL production. In addition, a segment of plasmid pLiz1 containing antimicrobial resistant genes, mobile genetic elements and insertion sequences is shown in Figure 1B . This is important since mobile genetic elements, which can sometimes be found on plasmids, are capable of disseminating antimicrobial resistance genes (Frost et al., 2005). Insertion sequences are a type of mobile genetic element which can aid in the transfer of antimicrobial resistance genes between the bacterial chromosome and a plasmid (Baquero et al., 2019). pLiz1 also appears to be conjugative since it contains 22 genes encoding various conjugative transfer proteins.

## Methods

Animals and isolation of antimicrobial resistant bacteria

Bingo was born at the Great Plains Zoo Feb 28, 2022. The fecal samples from Bingo were collected over a six-week period of time (July 27 to August 31, 2022). Upon shipping, all samples were stored in a -40℃ freezer until used for experiments. The diet of Bingo was wild herbivore plus plus, rhino browser cubes, ADF-16, beet pulp shreds, fruit or vegetable and alfalfa. 

Bacteria from the six fecal sample from Bingo were inoculated onto HardyCHROM™ ESBL agar plates (Hardy Diagnostics, Santa Maria, CA, USA). After incubating aerobically at 30 °C for 48 h pink bacterial colonies were subcultured onto trypticase soy agar plates (Hardy Diagnostics, Santa Maria, CA, USA). Bacterial colonies which are pink to magenta when grown on HardyCHROM™ ESBL agar are a presumptive positive for ESBL-producing *E. coli* according to the manufacturer.

Genome sequencing

The Quick-DNA Fungal/Bacterial Miniprep Kit (Zymo Research) was used to isolate genomic DNA from five bacterial colonies. The Rapid Barcoding Kit 24 V14 (SQK-RBK114.24) (Oxford Nanopore Technologies) was used for library preparation. DNA sequencing was done using a PromethION 2 Solo (Oxford Nanopore Technologies). Flye v1.4.2 was used for genome assembly (Kolmogorov et al., 2019). Annotation was done using the Rapid Annotation using Subsystem Technology tool kit (RASTtk) (Brettin et al., 2015). This database is housed at the Bacterial and Viral Bioinformatics Resource Center (BV-BRC) (https://www.bv-brc.org/). This Whole Genome Shotgun project has been deposited at DDBJ/ENA/GenBank under the accession JBTVZU000000000. The version described in this paper is version JBTVZU010000000.

Bacterial identification

Whole genome sequences were submitted to pubMLST for bacterial identification (Jolley et al., 2018). To confirm the pubMLST results average nucleotide identity (ANI) was used (Yoon et al., 2017).

Antimicrobial susceptibility testing

The Microscan autoSCAN-4 System (Beckman Coulter, Brea, CA, USA) was used to determine the antimicrobial susceptibility using NM45 susceptibility panels. Antimicrobial breakpoints were based upon the FDA Susceptibility Test Interpretive Criteria (https://www.fda.gov/drugs/development-resources/fda-recognized-antimicrobialsusceptibility-test- interpretive- criteria). A more detailed explanation of the method can be found in the study by Smith et al., 2024.

ESBL detection

The production of ESBLs was confirmed using the NG-Test CTX-M MULTI. The manufacturer’s guidelines were followed (Hardy Diagnostics, Santa Maria, CA, USA).

Prediction databases

The Clermont Phylotyper (https://ezclermont.hutton.ac.uk/) was used to determine the phylotype. The BacWGSTdb database was used to determine the sequence type, and to predict the presence of plasmid replicons and antimicrobial resistant genes. The BV-BRC database was used to predict genes involved in conjugative transfer. Default settings were used for all databases.
